# Addendum: Pongrácz et al. Grumpy Dogs Are Smart Learners—The Association between Dog–Owner Relationship and Dogs’ Performance in a Social Learning Task. *Animals* 2021, *11*, 961

**DOI:** 10.3390/ani11102815

**Published:** 2021-09-27

**Authors:** Péter Pongrácz, Gabriella Rieger, Kata Vékony

**Affiliations:** Department of Ethology, ELTE Eötvös Loránd University, 1053 Budapest, Hungary; riegergabriella@gmail.com (G.R.); kata.vekony.kami@gmail.com (K.V.)

The authors would like to make an addendum to their published paper [1].

In the originally published version of this article, ethical information was omitted, so the authors wish to add the following information regarding the ethical approval process of the experimental protocols:

- The animal experimentation methodology was reviewed and approved by the Animal Welfare Committee of Eötvös Loránd University (reference number TTK/6859/1 (2021), certificate number ELTE-AWC-011/2021), and additionally by a higher governmental office (National Food Chain Safety Office of Hungary, reference number PEI/001/1491-4/2015).
- Furthermore, the authors retrospectively obtained the approval for the human ethical aspects of the informed consent form and the participant prospectus for the dog owners who participated with their dogs in the experiments (the approval is listed under the code ELTE MÁB 12/2020).
- The authors submitted all the above mentioned documents, along with a copy of the owner consent form and participant prospectus, to *Animals*.

The change does not affect the scientific results.

The rest of the manuscript does not need to be changed.
